# Competition–colonization trade-off can explain any observed abundances and assumed competitive hierarchies

**DOI:** 10.1073/pnas.2526827122

**Published:** 2025-12-05

**Authors:** Alan Hastings, Kevin McCann, Gabriel Gellner

**Affiliations:** ^a^Department of Environmental Science and Policy, University of California, Davis, CA 95616; ^b^Santa Fe Institute, Santa Fe, NM 87501; ^c^Department of Integrative Biology, University of Guelph, Guelph, ON N1G 2W1, Canada

**Keywords:** competition–colonization trade-off, metacommunities, coexistence theory, inverse method

## Abstract

The competition–colonization trade-off is a possible explanation for coexistence of species in a metacommunity context that has been intensively studied for decades. Nonetheless, questions about the ubiquity and generality of the mechanism remain. The outcome of the model, equilibrium species abundances, are relatively easy to measure. However, the input into the basic model, the competitive hierarchy and the colonization rates, are not easy to measure in the field. We propose an approach that starts with an observed equilibrium configuration. We show that for any assumed competitive hierarchy we can find a corresponding set of colonization rates that would produce the observed equilibrium. We also find a simple formula for the colonization rates in terms of the observed abundances. This approach both shows that any observed set of abundances can result from a competition–colonization trade-off, and provides a method for future analyses. Additionally, generalizations of our approach can apply to generalizations of the basic competition–colonization model that avoid any biologically questionable assumptions.

Understanding how diverse ecological communities are maintained has been one of the key overarching questions in ecology for decades. One long-standing explanation for the maintenance of highly diverse communities is the trade-off between colonization and competition (1–8), which can be thought of as one of the simplest models underlying the concept of metacommunities (9). There has been controversy over the degree to which the model applies and how realistic are the assumptions (6, 10). Nevertheless, the basic model continues to be explored theoretically yielding new results (8) and used as an explanation for the maintenance of high diversity in grasslands and other systems. For this case, and many others, applying the ideas in a recently developed inverse approach will provide new insights.

The long-standing model for competition–colonization trade-offs (1, 2, 8) is expressed in terms of the dynamics of the fraction of locations that are occupied by species i, $p_{i}\left(t\right)$, where $m_{i}$ and $e_{i}$ are species specific colonization rates and extinction rates,

[1] $$\begin{array}{c} \frac{dp_{i}}{\mathrm{dt}}=m_{i}p_{i}\left(1-\sum_{j=1}^{i}p_{j}\right)-\left(\sum_{j=1}^{i-1}m_{j}p_{j}\right)p_{i}-e_{i}p_{i}. \end{array}$$

The model formulation assumes a complete competitive hierarchy so each species i can settle on empty space, or space occupied by a species j with j<i that is lower in the hierarchy, with immediate replacement. Colonization is assumed to be proportional to the fraction of available habitat for each species times the fraction of habitat occupied by that species. Typically, the model has been used to look at equilibria, all of which are stable, which satisfy the equation

[2] $$\begin{array}{c} 0=m_{i}p_{i}\left(1-\sum_{j=1}^{i}p_{j}\right)-\left(\sum_{j=1}^{i-1}m_{j}p_{j}\right)p_{i}-e_{i}p_{i}. \end{array}$$

Previous analyses (1–3, 8) of this model have made use of the simple structure to find the positive stable equilibrium of the model. First specify the parameters in the model, the $m_{i}$, and $e_{i}$. (In some formulations, all the $e_{i}$ are taken equal, and in this case without loss of generality they can be assumed to be one.) By proceeding from the most competitively dominant species, species 1, and then through each species in order of competitive dominance, it is simple to find the equilibrium of this model. For each species, it is easy to check if it has a positive equilibrium. This approach has led to many interesting and important results.

However, this analysis still does not answer the simple question of whether an observed set of species abundances could be explained by competition–colonization trade-offs. Analyses of this model which have proceeded by choosing the parameters in a random way (8) have found that only a fraction, roughly half, of the species pool, are found at the stable equilibrium. Moreover, although the species abundances, the $p_{i}$’s in the model, are easy to measure, the parameters (the colonization rates $m_{i}$ and the order of the competitive hierarchy) are not. This leads essentially to the kind of question studied in ref. 11, which suggested inferring processes from the observation data of a metacommunity through simulations. In a recent study of food webs based on Lotka–Volterra models, we used an inverse approach (12) to study a related question. Similarly for the competition–colonization trade-off model, which can be written as a Lotka–Volterra model, the inverse approach of analyzing the model by specifying the easy to measure abundances and solving for the difficult to directly measure parameters is very useful. This approach also answers the question: Are there configurations of species abundances and hierarchies that could not be explained by a competition–colonization trade-off? Unlike the case of using the inverse approach to analyze a food web model, where only numerical solutions were possible, here we find a simple analytical solution.

## Analysis and Results

We calculate the parameters that produce any observation of abundances for any assumed competitive hierarchy. We show first the approach and then a surprisingly simple formula for the colonization rate of species i as a function of the observed abundances of that species and all species able to out-compete species i. Similarly to the approach for solving for abundances from parameters, the approach we take starts with the competitive dominant. We can assume that $e_{1}=1$ which sets a timescale and we find that

[3] $$\begin{array}{c} m_{1}=\frac{1}{1-p_{1}} \end{array}$$

Note that this shows that for any abundance $p_{1}$ there is a colonization rate (namely $m_{1}$) that produces the observed abundance.

Writing out the solution for the next species in the competitive hierarchy will illustrate how this can be done for an arbitrary number of species. We will first simplify by assuming that all extinction rates are the same, so $e_{i}=1$ for all i. Specify $p_{1}$ and $p_{2}$. Then straightforward algebra shows that

[4] $$\begin{array}{c} m_{2}=\frac{1}{\left(1-p_{1}\right)\left(1-p_{1}-p_{2}\right)}=m_{1}\frac{1}{1-p_{1}-p_{2}} \end{array}$$

Finally, one can use mathematical induction (see methods) to show that, for any given competitive hierarchy and species abundances, the following choice of colonization parameters will produce the corresponding equilibrium:

[5] $$\begin{array}{c} m_{i}=\frac{1}{\left(1-\sum_{j=1}^{i-1}p_{j}\right)\left(1-\sum_{j=1}^{i}p_{j}\right)} \end{array}$$

(Note that, as required in the basic model, $m_{i}>m_{i-1}$, meaning that the order of competitive hierarchy and colonization ability are reversed.) An alternate way of writing Eq. **5** is

[6] $$\begin{array}{c} m_{i}=m_{i-1}\frac{\left(1-\sum_{j=1}^{i-2}p_{j}\right)}{\left(1-\sum_{j=1}^{i}p_{j}\right)}, \end{array}$$

which may be useful for providing insights. Finally, observe that Eq. **4** is equivalent to the general formulas Eqs. **5** and **6** evaluated at i=2.

## Discussion

The mathematical result shows that given any set of abundances, there is a set of colonization rates that will produce those abundances for any choice of competitive hierarchy. Thus, perhaps surprisingly, this analysis shows that any observed set of species abundances can be explained by competition–colonization trade-off. Thus, no set of species abundances rules out competition–colonization trade-off as the explanation for coexistence, and no information about the hierarchy can be deduced from the abundances of the species. Thus the competitive dominant may be the rarest species or the most common species; no inferences about place in the competitive hierarchy can be made from species abundances which seems to contradict some earlier results (e.g., ref. 4).

There are a number of other simple conclusions about equilibria that can be drawn from the general approach and solution Eq. **5**. For example, the colonization rate of species i corresponding to an equilibrium abundance of species i increases as the observed abundance of species i increases. An increase in the observed abundance of species i at equilibrium not only increases the corresponding colonization rate of species i but also increases the corresponding colonization rates of all species lower in the competitive hierarchy.

Another important, and slightly more complex, general conclusion is possible. If there is a maximum possible colonization rate γ over all species, we can then find a bound for the maximum overall occupancy of the habitat. Define α as the fraction of habitat that is occupied by any species so 1−α is the fraction of empty habitat. Then starting with Eq. **5**, by replacing $\sum_{j=1}^{i-1}p_{j}$ by $\sum_{j=1}^{i}p_{j}$ we can easily see that $\gamma >{\left(1-\alpha \right)}^{-2}$. (Note that the inequality should be quite close to an equality since the only difference from an equality is due to the abundance of the best colonizer.) This inequality can be rearranged to show that

[7] $$\begin{array}{c} \alpha <1-\sqrt{\left(1/\gamma \right)}. \end{array}$$

Equivalently, we have shown that the fraction of patches that are unoccupied is greater than $\sqrt{\left(1/\gamma \right)}$. Since it is reasonable to assume there is maximum colonization rate γ, this establishes an overall maximum possible habitat occupancy rate α. If the overall occupancy rate is higher, then the simple competition–colonization model cannot apply, and a different explanation is required, perhaps relaxing assumptions as in, for example, ref. 6.

There is an interesting comparison between space occupancy by a single species by itself and by a metacommunity. A single species with colonization rate γ would have the fraction of unoccupied patches at 1/γ, while if that species was the best colonizer and worst competitor in a metacommunity the fraction of unoccupied area can be close to $\sqrt{\left(1/\gamma \right)}$. Since $\sqrt{\left(1/\gamma \right)}\gg 1/\gamma$, adding species that are poorer colonizers but better competitors reduces overall space occupancy.

One recent careful study (7) looking at the competition–colonization trade-off in trematodes could serve as a platform for more detailed examination using the results here. That study had both determinations of the competitive hierarchy and the species abundances. Their conclusion was that while the competition–colonization trade-off was at least a partial explanation for the observed abundances, other factors would be needed. The results we have could be used to demonstrate that competition–colonization would be sufficient, but if other biological considerations are required, a more general model would be needed.

The inverse approach we describe here can also apply to generalizations of the basic competition–colonization trade-off model, but the conclusions are not quite so clear-cut. This is important since many communities do not meet the assumptions for the basic model as is easily seen in microbial communities (13). For the basic model (1, 2), the simple formula here provides clear and simple conclusions. Generalizations (e.g., ref. 6) no longer have a strict hierarchy, but still fall in the class of Lotka–Volterra competition models. For these models, the inverse approach can still be used as in our earlier work (12). However, as there are more parameters than species abundances, this solution is underdetermined, meaning that there is not a unique solution. Yet, it is clear once again that any set of abundances can be produced. In fact, if we assumed a complete hierarchy, we could produce the observed abundances, so the abundances could be produced under the more general model. The inverse approach used in ref. 12 can be used to reach further conclusions.

A sharp contrast with the earlier work (12) for the general Lotka–Volterra model is that for the competition–colonization model any positive equilibrium is stable, while for the general Lotka–Volterra model that is not true. However, as conjectured in ref. 6, and as supported by numerical results, for the generalization of the competition–colonization model used in ref. 6, any feasible equilibrium is stable. So, once again, as in the simple version with the fixed hierarchy, only very limited conclusions about whether the competition–colonization trade-off is shown or not can be drawn from abundances. The ideas developed here can potentially be combined with different approaches (14) for testing the theory.

Since the generalizations of the approach developed here can apply to many of the generalizations of the basic (1, 2) model, there is a great opportunity to use the approach presented here for future work. Thus, the basic model can still serve as a way to emphasize the importance of trade-offs and as a framework for more detailed investigations. Our work also emphasizes that more information than equilibrium observed abundances would be needed to demonstrate that the basic model does not apply. Adding in other considerations, including stochasticity and temporal dynamics, and the role of other kinds of interactions, will provide new insights into the more general and burgeoning field of metacommunities (9).

## Methods

Here, we go through the proof by mathematical induction of the general formula Eq. **5**. We need to show that the formula Eq. **5** holds for i=n if it holds for i=n−1. We can compute directly from Eq. **2** that

[8] $$\begin{array}{c} m_{i}=\frac{1+\sum_{j=1}^{i-1}m_{j}p_{j}}{1-\sum_{j=1}^{i}p_{j}} \end{array}$$

As noted in the main text, Eq. **4** shows that Eq. **5** holds for i=1 and i=2. Thus we assume that Eq. **5** holds for i=n−1. We need to show that this implies that, for i=n, $m_{i}$ calculated directly, Eq. **8**, is equal to the general formula for $m_{i}$, Eq. **5**, namely that

[9] $$\begin{array}{c} \frac{1+\sum_{j=i}^{i-1}m_{j}p_{j}}{1-\sum_{j=1}^{i}p_{j}}=\frac{1}{\left(1-\sum_{j=1}^{i-1}p_{j}\right)\left(1-\sum_{j=1}^{i}p_{j}\right)} \end{array}$$

We start by rearranging and breaking out the terms corresponding to the assumed n−1 case, writing Eq. **9** as

[10] $$\begin{array}{c} 0=\left(\sum_{j=1}^{n-2}p_{j}m_{j}\right)+p_{n-1}m_{n-1}-\left(\sum_{j=1}^{n-2}p_{j}\right)-p_{n-1}\\ -\left(\sum_{j=1}^{n-2}p_{j}+p_{n-1}\right)\left(\sum_{j=1}^{n-2}p_{j}m_{j}+p_{n-1}m_{n-1}\right) \end{array}$$

Removing terms that cancel as implied by assuming Eq. **5** holds for i=n−1, we need to show that

[11] $$\begin{array}{c} 0=p_{n-1}m_{n-1}-p_{n-1}-\left(\sum_{j=1}^{n-2}p_{j}\right)p_{n-1}m_{n-1}\\ -\left(\sum_{j=1}^{n-2}p_{j}m_{j}\right)p_{n-1}-p_{n-1}p_{n-1}m_{n-1} \end{array}$$

Simplifying and rearranging we get

[12] $$\begin{array}{c} m_{n-1}=\frac{1+\sum_{j=1}^{n-2}m_{j}p_{j}}{1-\sum_{j=1}^{n-1}p_{j}} \end{array}$$

As this is equivalent to Eq. **8** obtained by direct computation evaluated at i=n−1, we have the desired result.

## Data Availability

There are no data underlying this work.
